# Prevalence of Hyperuricemia Among Chinese Adults: Findings From Two Nationally Representative Cross-Sectional Surveys in 2015–16 and 2018–19

**DOI:** 10.3389/fimmu.2021.791983

**Published:** 2022-02-07

**Authors:** Mei Zhang, Xiaoxia Zhu, Jing Wu, Zhengjing Huang, Zhenping Zhao, Xiao Zhang, Yu Xue, Weiguo Wan, Chun Li, Wenrong Zhang, Linhong Wang, Maigeng Zhou, Hejian Zou, Limin Wang

**Affiliations:** ^1^National Center for Chronic and Noncommunicable Disease Control and Prevention, Chinese Center for Disease Control and Prevention, Beijing, China; ^2^Division of Rheumatology, Huashan Hospital, Fudan University, Shanghai, China

**Keywords:** hypeuricemia, prevalence, trends, Chinese, gout

## Abstract

**Objective:**

To determine the nationwide prevalence of hyperuricemia in China and evaluate its trends and associated risk factors.

**Methods:**

Using a multi-stage, stratified, cluster-randomized sampling design, two cross-sectional surveys (representative of national and provincial information) were conducted in 31 provinces (autonomous regions and municipalities) in mainland China, with 166, 861 Chinese adults in 2015–16 and 168, 351 in 2018–19. Serum uric acid (SUA) levels of all participants were measured after a >10-hour overnight fast. Hyperuricemia (HUA) was defined when SUA was >420 μmol/L. Prevalence estimates were weighted to represent the total population considering the complex sampling design. Multivariable logistic regression models was used to estimate factors associated with HUA.

**Results:**

The overall hyperuricemia prevalence in the Chinese adult population was 11.1% (95% confidence interval 10.3% to 11.8%) in 2015–16 and 14.0% (13.1% to 14.8%) in 2018–19; an alarming rise was observed in the three years. Hyperuricemia was more common in men with 19.3% (17.9% to 20.7%) in 2015–16 and 24.4% (23.0% to 25.8%) in 2018–19, although the prevalence also escalated from 2.8% (2.5% to 3.0%) in 2015–16 to 3.6% (3.2% to 4.0%) in 2018–19 in women. The hyperuricemia risk factors include the urban culture, settlement in the East, Zhuang descent, high education, heavy or frequent beer drinking, high red meat intake, physical inactivity, high body mass index, central obesity, hypertension, hyperlipidemia, and low glomerular filtration rate.

**Conclusion:**

The estimated hyperuricemia prevalence among Chinese adults was 14.0% in 2018-19; significant escalating trends were observed between 2015-16 and 2018-19.

## Introduction

Hyperuricemia (HUA) has gradually become an important worldwide public health issue (1). HUA is critically involved in the development of gout (2), and elevated serum uric acid (SUA) is associated with increased risks of onset and progression of chronic kidney disease, end-stage kidney disease (3, 4), cardiovascular events, and death (5, 6). People with high SUA have shown an increased risk of readmission for heart failure and longer hospital stays (7). A national health and nutrition examination survey in the United States revealed similar mortality risks for HUA and diabetes (8).

The prevalence of HUA among Chinese adults in 2009–2010 was 8.4% (9). Miao et al. reported a significantly higher HUA prevalence (18.32% in men; 8.56% in women) in coastal cities in 2008 (10), and a recent study showed HUA prevalence of 11.3% (20.7% in men; 5.6% in women) in the eastern Chinese population (21 cities from the five provinces of Shanghai, Zhejiang, Jiangsu, Anhui, and Jiangxi) in 2014-15 (11). According to a recent meta-analysis, the HUA prevalence has been increasing steadily in China in the past few decades (12). However, large-scale national population-based data is lacking in China.

In this study, data from two representative surveys, presenting national and provincial data, conducted from 2015–16 and 2018–19, were researched to determine the nationwide prevalence of HUA in China and evaluate its trends and associated risk factors.

## Methods

### Study Population

The Chinese Chronic and Risk Factor Surveillance (CCDRFS) is the national system that conducts health-related surveys on Chinese adults regarding chronic and noncommunicable diseases and their risk factors. Established in 2004, CCDRFS has conducted six field surveys so far; four in 2004, 2007, 2010, and 2013 and the other two, the most recent ones, in 2015 and 2018 within the Disease Surveillance Points (DSPs) system covered 31 province (autonomous regions and municipalities) (**Supplementary 1** and **Supplementary Figure 1**). Since CCDRFS 2013, 298 surveillance counties/districts were randomly selected from the DSPs system (13, 14). These surveys were designed to represent both national and provincial data. The target population included adults aged 18 years and above and living in mainland China. The residents who met the following inclusion criteria were included: aged 18 years or older; having lived in the address for more than 6 months in the past 12 months; not pregnant; not having a serious health condition or illness that prevents from participating, including intellectual disability or language disorder. The ethical review committee of the Chinese Center for Disease Control and Prevention (China CDC) approved the CCDRFS 2015 (approval number 201519-B) and that of the National Center for Chronic and Noncommunicable Disease Control and Prevention, China CDC approved the CCDRFS 2018 (approval number 201819). All participants of the CCDRFS study consented in writing.

### Sampling Methods

**Figure 1:**
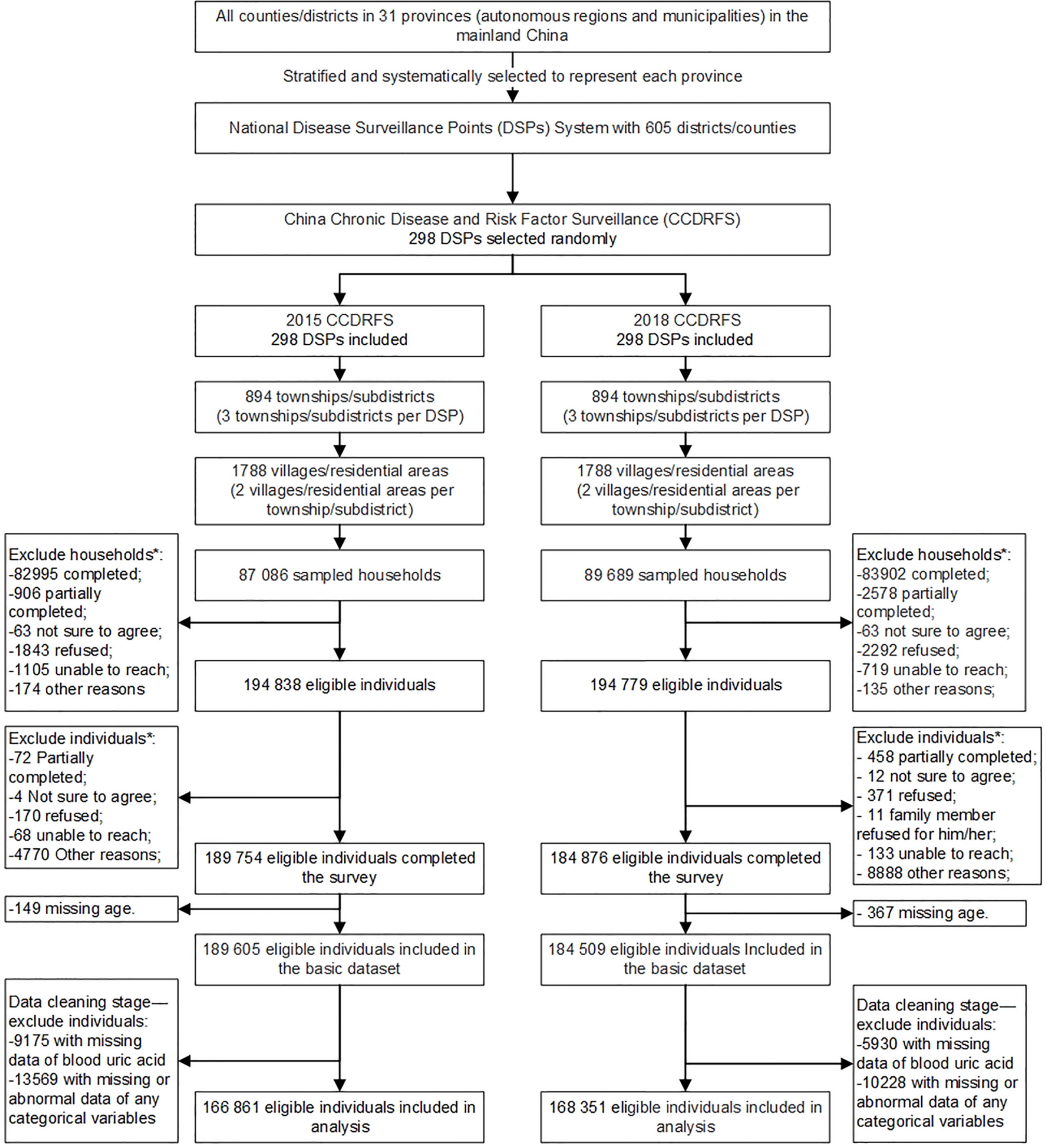
Flow diagram of study sample.

The CCDRFS 2015 and CCDRFS 2018 used a complex multi-stage cluster sampling method to select eligible participants within every surveillance district/county for every survey. First, three townships or subdistrict were selected using the systematic sampling method. Second, two administrative villages or communities were selected using the same sampling technique as in each chosen township/subdistrict. Third, each administrative village or community was divided into several residential quarters, each with nearly 60 households. Finally, 45 households from one residential quarter were selected to be the target households. The selected households and eligible family members were invited to participate in the survey. Both of two surveys started on Auguest and by June of the next year. In total, 82 995 of the 87 086 households in 2015–16 and 83 902 of the 89 689 households in 2018–19 completed the survey, giving a 94.4% family response rate. Of 389 617 eligible participants, 374 630 completed the interview, giving an individual response rate of 96.2%. **Figure 1** shows the detailed sampling frames.

### Data Collection

Each field survey started in August of the survey year, with most interviews and exams finished in the same year. All remaining visits were completed by June of the next year. In both surveys, a householder or adult who knew the details of the household well was interviewed using a questionnaire. The economic information of the household and basic information of all family members (e.g., birth date, sex, and name) were collected. Participants who met the inclusion criteria were given individual questionnaires to obtain detailed information on demographic characteristics, lifestyle factors, and the history of chronic diseases. Smoking status was obtained using the Global Adult Tobacco Survey questionnaire. The Global Physical Activity Questionnaire was used to assess physical activity. Dietary behavior in the preceding 12 months was evaluated using a food frequency questionnaire. The participants were also invited to a community health service station for measuring physical attributes and collecting biological samples. Weight and height were measured using a standard protocol, and body mass index (BMI) was calculated by dividing the weight in kilograms by the square of height in meters. Waist circumference was measured in the standing position, midway between the lower edge of the costal arch and upper edge of the iliac crest. Systolic and diastolic blood pressures were measured three times, with one-minute intervals in between, using an electronic sphygmomanometer (HBP-1300; OMRON Healthcare Product Development Dalian Co., Ltd., Dalian, China) after the participants had rested for 5 minutes in a seated position. Therefore, each parameter was recorded three times and the average of the last two readings was used for data analysis. Blood samples were collected in the morning, after an overnight fast of ≥10 hours. In 2018-19, an oral glucose tolerance test (OGTT) was also performed; the participants without a self-reported history of diabetes were given a standard 75 g glucose solution, and plasma glucose levels were measured at 0 and 2 hours after its administration. Besides, in 2018-19, the participants were asked to collect samples of their first-morning urine at home and submit them to the interviewers when they arrived at the health service station. After pretreatment at the health service station, the plasma, serum, and urine samples were transported at 4°C within 2 h of pretreatment to the laboratories of the local Centers for Disease Control and Prevention (CDCs), and stored in a deep freezer or dry ice at −20 or −80°C, respectively. Plasma glucose was detected within 48 h after sample collection. Within one month of collection, all serum and urine samples frozen at −80°C with dry ice were shipped by air to the central laboratories. The uric acid, total cholesterol (TC), triglycerides (TG), and creatinine in serum, and creatinine and microalbumin in urine, were tested using an automatic biochemical analyzer. **Supplementary Table 1** shows the details of the methods and equipment used in the two surveys to test for biochemical indicators. Trained interviewers from the local CDCs carried out all the interviews and measurements and collected biochemical samples. All local labs were certified for quality before the analysis, and the quality was maintained and validated through daily quality checks. The central labs were certified by the College of American Pathologists and followed stringent quality control procedures for all tests.

### Definition of HUA and Related Factors

Given that the level at which uricemia becomes abnormal is still disputed, we have defined HUA according to the commonly used cut-off point of 420 μmol/L, for both men and women. Besides, for HUA in women, we exclusively used a sex-specific cut-off of 360 μmol/L (9). The participants were categorized into six groups based on age: 18–29, 30–39, 40–49, 50–59, 60–69, and ≥70 years. The neighborhood committees and villages were considered as urban and rural areas, respectively. According to the geographical location, the 31 provinces (autonomous regions and municipalities) were classified into three regions: the East, Middle, and the West (**Supplementary Figure 1**). Participants were divided into the following groups based on educational attainment: those who finished primary school or less, those who finished secondary school, those who finished high school, and those who finished college or above. Seven ethnic groups were presented: Han, Hui, Manchu, Tibetan, Uighur, Zhuang, and others (including 50 ethnic minority groups). Household per capita income was classified into four groups with tertile, and participants who refused to provide or did not know the income was categorized as a separate group. Participants who were smoking during the survey period were defined as smokers. Male participants who drank pure alcohol ≥25 g/d and female participants who drank pure alcohol ≥15 g/d were defined as heavy drinkers (15). Insufficient intake of vegetables and fruits and physical inactivity were identified based on WHO definitions (16). According to standard WHO criteria, overweight was defined by a BMI of 25.0 to 29.9 kg/m^2^, and obesity by a BMI of 30.0 kg/m^2^ or higher. Having a waist circumference of 90 cm or more in men and 85 cm or more in women was defined as central obesity. Hypertension was diagnosed when the systolic blood pressure was ≥140 mmHg, diastolic blood pressure was ≥90 mmHg, or the participants were taking medications for the treatment of high blood pressure during the survey period (17). High total cholesterol (TC) was defined by TC ≥ 6.22 mmol/L and high low-density lipoprotein cholesterol (LDL-C) was defined by LDL-C ≥ 4.14 mmol/L (18). Among participants without a self-reported diabetes history before the survey, those with fasting blood glucose ≥7.0 mmol/L or/and OGTT glucose ≥11.1 mmol/L were regarded as participants with newly diagnosed diabetes, and those with fasting blood glucose in the range of 6.1–6.9 mmol/L or/and OGTT glucose in the range of 7.8–11.0 mmol/L were regarded as prediabetic (19). The estimated glomerular filtration rate (eGFR) was considered low when the eGFR was <60 ml/min*1·73m^2^. Albuminuria was defined as urine microprotein/creatinine >30 mg/g (20).

### Statistical Analysis

We excluded 24 313 participants with missing or abnormal values for demographic or socio-economic characteristics and 15105 participants with missing or abnormal blood uric acid data. Therefore, a total of 335 212 individuals (166 861 from CCDRFS 2015 and 168 351 from CCDRFS 2018) were included in this analysis. We described the sample size of the study population and the general characteristics of Chinese adults aged 18 years and above. All estimates, including those of prevalence, proportions, and means, were adjusted for age based on China’s 2010 census released by the National Bureau of Statistics. Logistic regression models were used to study the linear trends in the prevalence of hyperuricemia over the survey years. The chi-square test was used to compare prevalence estimates between groups (e.g., divided based on residential status, smoking history, and hypertension history), and logistic regression models were used to examine the trends of ordered categorical variables (e.g., age, education attainment, and income). Based on mixed data from two surveys, survey, multiariable logistic regression was used to examine the association of the odds of hyperuricemia with potential risk factors, including demographic characteristics, behaviro habits, and chronic conditions. Taylor series linearization method with a finite population correction was used to estimate standard errors (SE), accounting for the complex sampling design. All tests were two-sided, and a *P*-value < 0.05 was considered statistically significant. All analyses were performed using SAS 9.4 (SAS Institute, Inc., Cary, North Carolina, USA). Choropleth maps were generated using the R software (version 3.6.1).

### Role of the Funding Source

The funding bodies had no role in the study design, data collection, data analysis, data interpretation, or manuscript preparation. All of the authors had full access to all the data in the study and the corresponding authors accept final responsibility to submit for publication.

## Results

The general characteristics of the study population and distribution of potential risk factors are shown in **Table 1**. A total of 166,861 and 168,351 adults were included in the surveys of 2015–16 and 2018–19, respectively. The weighted mean age was 43.9 (SD 16.0) years in the 2015–16 survey and 43.7 (SD 16.1) in the 2018–19 survey. Compared to those in 2015–16, the adults in 2018–19 were better educated, earned a higher income, consumed red meat more commonly, were physically inactive, and had higher TC and TG levels.

**Table 1:** General Characteristics of Samples in Two Surveys*.

	Men		Women		Overall	
	2015-16 (n = 77480)	2018-19 (n = 74184)	2015-16 (n = 89381)	2018-19 (n = 94167)	2015-16 (n = 166861)	2018-19 (n = 168351)
Age group (years), *n (%)*						
18-29	6356 (8.2)	3707 (5.0)	8045 (9.0)	5025 (5.3)	14401 (8.6)	8732 (5.2)
30-39	8834 (11.4)	6743 (9.1)	11114 (12.4)	9838 (10.4)	19948 (12.0)	16581 (9.8)
40-49	16420 (21.2)	12666 (17.1)	20403 (22.8)	17767 (18.9)	36823 (22.1)	30433 (18.1)
50-59	18762 (24.2)	18850 (25.4)	22182 (24.8)	25618 (27.2)	40944 (24.5)	44468 (26.4)
60-69	18056 (23.3)	21104 (28.4)	19166 (21.4)	24662 (26.2)	37222 (22.3)	45766 (27.2)
≥70	9052 (11.7)	11114 (15.0)	8471 (9.5)	11257 (12.0)	17523 (10.5)	22371 (13.3)
Urban, *n (%)*	30677 (39.6)	28859 (38.9)	37731 (42.2)	39727 (42.2)	68408 (41.0)	68586 (40.7)
Location, *n (%)*						
East	29115 (37.6)	27534 (37.1)	33888 (37.9)	35811 (38.0)	63003 (37.8)	63345 (37.6)
Middle	22718 (29.3)	20984 (28.3)	25969 (29.1)	27193 (28.9)	48687 (29.2)	48177 (28.6)
West	25647 (33.1)	25666 (34.6)	29524 (33.0)	31163 (33.1)	55171 (33.1)	56829 (33.8)
Education, *n (%)*						
Primary school or less	32173 (41.5)	30915 (41.7)	49609 (55.5)	52840 (56.1)	81782 (49.0)	83755 (49.8)
Secondary school	27514 (35.5)	26516 (35.7)	23506 (26.3)	24528 (26.0)	51020 (30.6)	51044 (30.3)
High school	11794 (15.2)	11409 (15.4)	9821 (11.0)	10511 (11.2)	21615 (13.0)	21920 (13.0)
College or higher	5999 (7.7)	5344 (7.2)	6445 (7.2)	6288 (6.7)	12444 (7.5)	11632 (6.9)
Ethnicity, *n (%)*						
Han	68607 (92.4)	65275 (88.0)	78915 (88.3)	82805 (87.9)	147522 (88.4)	148080 (88.0)
Hui	987 (0.5)	1075 (1.4)	1228 (1.4)	1366 (1.5)	2215 (1.3)	2441 (1.4)
Manchu	979 (1.2)	974 (1.3)	1141 (1.3)	1304 (1.4)	2120 (1.3)	2278 (1.4)
Tibetan	1012 (1.3)	1397 (1.9)	1342 (1.5)	1627 (1.7)	2354 (1.4)	3024 (1.8)
Uighur	1119 (1.4)	903 (1.2)	1091 (1.2)	1174 (1.2)	2210 (1.3)	2077 (1.2)
Zhuang	748 (1.0)	917 (1.2)	955 (1.1)	1311 (1.4)	1703 (1.0)	2228 (1.3)
Others	4028 (5.2)	3643 (4.9)	4709 (5.3)	4580 (4.9)	8737 (5.2)	8223 (4.9)
Income per capita (CNY), *n (%)*						
Q1 (<¥6000)	17204 (22.2)	14681 (19.8)	18418 (20.6)	17164 (18.2)	35622 (21.3)	31845 (18.9)
Q2 (¥6000-11999)	15549 (20.1)	13329 (18.0)	17750 (19.9)	16104 (17.1)	33299 (20.0)	29433 (17.5)
Q3 (¥12000-23999)	17491 (22.6)	15050 (20.3)	20558 (23.0)	19323 (20.5)	38049 (22.8)	34373 (20.4)
Q4 (≥¥24000)	14521 (18.7)	15673 (21.1)	17253 (19.3)	20471 (21.7)	31774 (19.0)	36144 (21.5)
Refused/Don’t know	12715 (16.4)	15451 (20.8)	15402 (17.2)	21105 (22.4)	28117 (16.9)	36556 (21.7)
Current smoking, *n (%)*	40884 (52.8)	37841 (51.0)	2642 (3.0)	2627 (2.8)	43526 (26.1)	40468 (24.0)
Heavy drinking, *n (%)*	15745 (20.3)	13172 (17.8)	4231 (4.7)	1327 (1.4)	19976 (12.0)	14499 (8.6)
Beer drinking, *n (%)*						
Never	53902 (69.6)	57113 (77.0)	82836 (92.7)	88517 (94.0)	136738 (81.9)	145630 (86.5)
At least once a year	13779 (17.8)	9683 (13.1)	5386 (6.0)	4614 (4.9)	19165 (11.5)	14297 (8.5)
At least once a week	9799 (12.6)	7388 (10.0)	1159 (1.3)	1036 (1.1)	10958 (6.6)	8424 (5.0)
Fruit/vegetable intake <400g/d, *n (%)*	42127 (54.4)	35319 (47.6)	48289 (54.0)	43688 (46.4)	90416 (54.2)	79007 (46.9)
Red meat intake ≥ 100g/d, *n (%)*	23561 (30.4)	31229 (42.1)	18777 (21.0)	29353 (31.2)	42338 (25.4)	60582 (36.0)
Physical inactivity (<150min/w), *n (%)*	15670 (20.2)	16276 (21.9)	14654 (16.4)	16683 (17.7)	30324 (18.2)	32959 (19.6)
BMI group (kg/m^2^), *n (%)*						
<18.5	2720 (3.5)	2164 (2.9)	3470 (3.9)	2819 (3.0)	6190 (3.7)	4983 (3.0)
18.5-24.9	45399 (58.6)	41001 (55.3)	51300 (57.4)	51418 (54.6)	96699 (58.0)	92419 (54.9)
25.0-29.9	25087 (32.4)	26228 (35.4)	28466 (31.8)	32685 (34.7)	53553 (32.1)	58913 (35.0)
≥30.0	4274 (5.5)	4791 (6.5)	6145 (6.9)	7245 (7.7)	10419 (6.2)	12036 (7.1)
Central obesity, *n (%)*	23410 (30.2)	27369 (36.9)	30794 (34.5)	39639 (42.1)	54204 (32.5)	67008 (39.8)
Hypertension, *n (%)*	32309 (41.7)	32429 (43.7)	33928 (38.0)	37145 (39.4)	66237 (39.7)	69574 (41.3)
High TC, *n (%)*	5077 (6.6)	6819 (9.2)	7537 (8.4)	11331 (12.0)	12614 (7.6)	18150 (10.8)
High TG, *n (%)*	13743 (17.7)	15288 (20.6)	12316 (13.8)	16696 (17.7)	26059 (15.6)	31984 (19.0)
Glycemia status, *n (%)*						
Normal	NA	42288 (58.7)	NA	56177 (62.1)	NA	98465 (60.6)
Prediabetes	NA	16772 (23.3)	NA	19501 (21.6)	NA	36273 (22.3)
Newly diagnosed diabetes	NA	7799 (10.8)	NA	7778 (8.6)	NA	15577 (9.6)
Diagnosed diabetes	NA	5148 (7.1)	NA	7000 (7.7)	NA	12148 (7.5)
Low eGFR, *n (%)*	NA	3073 (4.3)	NA	3528 (3.9)	NA	6601 (4.1)
Albuminuria, *n (%)*	NA	5999 (8.3)	NA	8582 (9.5)	NA	14581 (9.0)

*Percentages may not sum to 100 due to rounding. NA, data not available; BMI, body mass index; TC, total cholesterol; TG, triglycerides; eGFR, estimated glomerular filtration rate. ¥ 100, £11; €13; $15.5.

**Figure 2:**
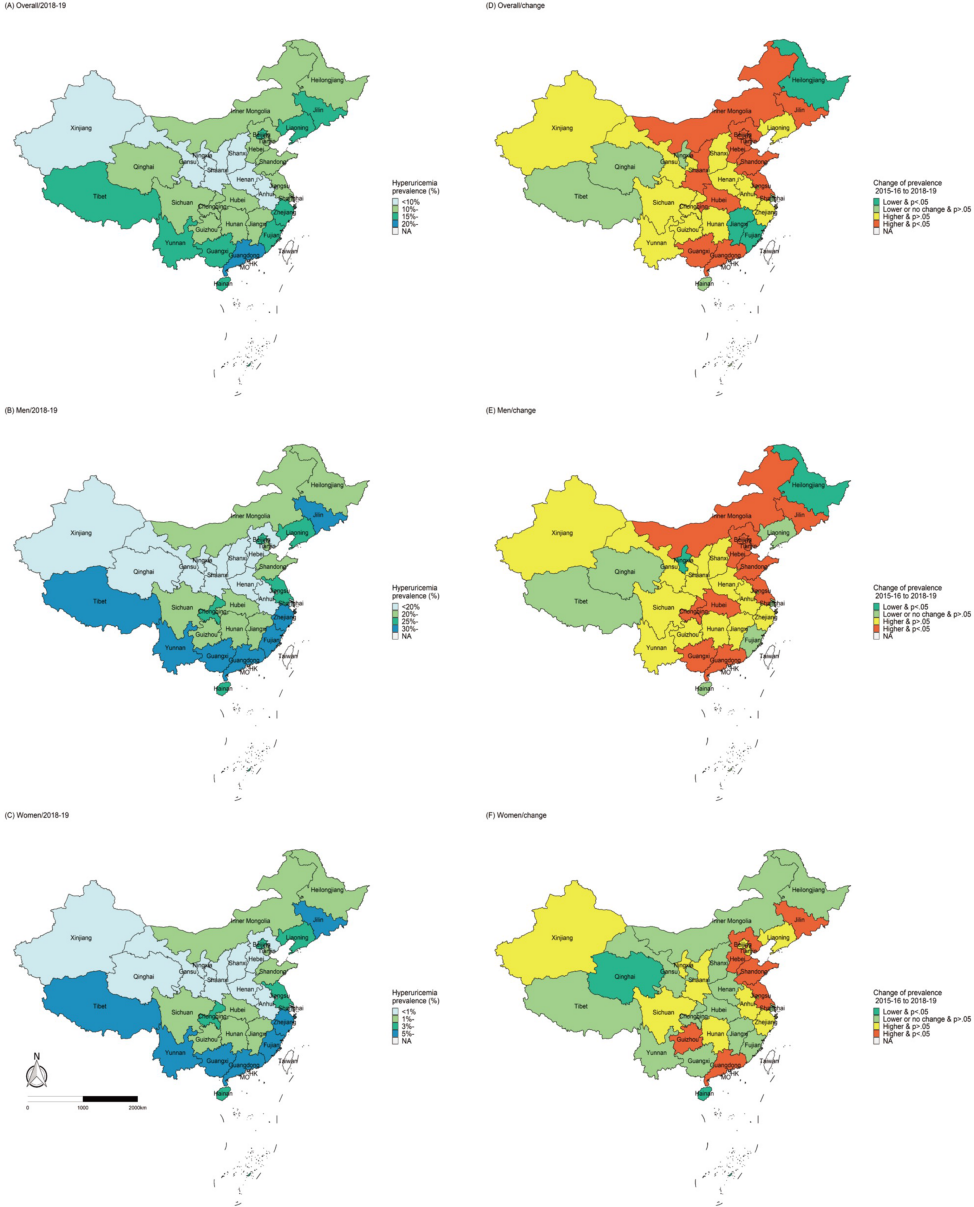
Geographic distribution of hyperuricemia prevalence in 2018–19 and changes from 2015–16 to 2018–19.

In 2018–19, the weighted prevalence of HUA was 14.0% (95% CI, 13.1–14.8%) in Chinese adults, with a higher estimate in men (24.4% [95% CI, 23.0–25.8%]) than in women (3.6% [95% CI, 3.2–4.0%]) (*P*<0·001). Significant age-based differences were also observed. The prevalence was the highest at 32.3% in the 18–29 years old group and then decreased with age; the lowest was at 17.0% in men in the 60–69 years old group. However, in women, the prevalence decreased in the childbearing age and increased after menopause. Besides, the prevalence increased with education and income; it was 19.0% in adults who had high education (finished college or above) and 16.9% in those with the highest income. Comparing the major ethnic groups, the HUA prevalence was the highest in Zhuang (17.1% [95% CI, 14.3–20.0%] and the lowest in Uighur (2.1% [95% CI, 0.6–3.0%]). The prevalence also differed significantly based on geography and human settlement; it was higher in the urban areas, Eastern China, and the coastal provinces (**Table 2** and **Figure 2**). And the prevalence was also higher in participants with specific health-related risk factors (e.g., smoking, heavy drinking, high red meat intake, physical inactivity, overweight or obesity, etc.; **Table 3**).

**Table 2:** Prevalence of hyperuricemia among adult in mainland China by characteristics, 2015-16 and 2018-19*.

Characteristics	Prevalence, % (95%CI)								
	Men			Women*^§^*			Overall		
	2015-16	2018-19	Changes of prevalence, 2018-19 vs 2015-16	2015-16	2018-19	Changes of prevalence, 2018-19 vs 2015-16	2015-16	2018-19	Changes of prevalence, 2018-19 vs 2015-16
Overall	19.3 (17.9,20.7)	24.4 (23.0,25.8)	5.1 (4.4,5.8)*^†^*	2.8 (2.5,3.0)^‡^	3.6 (3.2,4.0)^†^	0.8 (0.6,1.1)*^†^*	11.1 (10.3,11.8)	14.0 (13.1,14.8)	2.9 (2.5,3.3)*^†^*
Age group (years)									
18-29	23.9 (21.7,26.1)	32.3 (29.4,35.3)	8.4 (6.7,10.1)*^†^*	2.7 (2.2,3.1)	4.2 (3.2,5.2)	1.5 (0.9,2.1)*^†^*	13.4 (12.2,14.6)	18.0 (16.1,19.8)	4.6 (3.7,5.6)*^†^*
30-39	20.6 (19.1,22.1)	28.4 (26.0,30.7)	7.8 (6.6,8.9)*^†^*	1.7 (1.3,2.0)	2.3 (1.8,2.9)	0.7 (0.3,1.0)	11.3 (10.4,12.1)	15.5 (14.2,16.9)	4.3 (3.6,4.9)*^†^*
40-49	18.6 (16.1,21.1)	21.4 (20.0,22.9)	2.8 (1.5,4.1)*^†^*	2.1 (1.7,2.5)	2.2 (1.9,2.5)	0.1 (-0.2,0.3)	10.4 (9.0,11.7)	11.8 (11.1,12.5)	1.4 (0.7,2.1)
50-59	15.4 (14.4,16.5)	18.1 (17.0,19.3)	2.7 (1.9,3.5)*^†^*	2.8 (2.5,3.1)	3.7 (3.2,4.1)	0.9 (0.6,1.2)*^†^*	9.2 (8.6,9.7)	10.9 (10.2,11.6)	1.8 (1.3,2.2)*^†^*
60-69	15.0 (13.9,16.0)	17.0 (15.9,18.0)	2.0 (1.2,2.7)*^†^*	4.0 (3.5,4.4)	4.4 (4.0,4.8)	0.4 (0.1,0.7)	9.5 (8.9,10.2)	10.7 (10.1,11.4)	1.2 (0.7,1.7)*^†^*
≥70	17.0 (15.3,18.7)	19.5 (18.2,20.7)	2.4 (1.3,3.5)*^†^*	6.3 (5.5,7.2)	8.0 (7.0,9.1)	1.7 (0.9,2.4)*^†^*	11.3 (10.2,12.4)	13.4 (12.4,14.4)	2.1 (1.2,2.9)*^†^*
* P* for trend	<0.0001	<0.0001		<0.0001	<0.0001		<0.0001	<0.0001	
Residence									
Urban	22.5 (20.4,24.6)	28.1 (26.1,30.2)	5.7 (4.6,6.8)*^†^*	2.9 (2.6,3.3)	4.1 (3.4,4.7)	1.1 (0.7,1.5)*^†^*	12.8 (11.7,13.9)	16.1 (14.8,17.3)	3.3 (2.7,3.9)*^†^*
Rural	16.0 (15.1,16.9)	20.4 (19.1,21.8)	4.4 (3.7,5.2)*^†^*	2.6 (2.3,2.9)	3.1 (2.8,3.5)	0.5 (0.3,0.8)*^†^*	9.3 (8.7,9.8)	11.7 (10.9,12.5)	2.4 (2.0,2.9)*^†^*
* P* for difference	<0.0001	<0.0001		0.1316	0.0099		<0.0001	<0.0001	
Location									
East	21.5 (18.9,24.1)	29.6 (27.0,32.2)	8.1 (7.0,9.3)*^†^*	2.8 (2.5,3.1)	4.8 (4.0,5.6)	2.0 (1.6,2.5)*^†^*	12.2 (10.8,13.6)	17.1 (15.5,18.8)	5.0 (4.3,5.6)*^†^*
Middle	16.3 (14.7,17.9)	18.4 (17.0,19.8)	2.1 (1.2,3.0)*^†^*	2.6 (2.2,3.0)	2.6 (2.2,3.0)	0.0 (-0.2,0.3)	9.4 (8.5,10.2)	10.4 (9.7,11.2)	1.0 (0.5,1.5)*^†^*
West	19.5 (17.9,21.1)	23.1 (21.2,25.0)	3.6 (2.4,4.8)*^†^*	3.0 (2.4,3.6)	2.8 (2.4,3.3)	-0.2 (-0.5,0.2)	11.4 (10.4,12.3)	13.0 (12.0,14.1)	1.7 (1.1,2.3)*^†^*
* P* for difference	<0.0001	<0.0001		0.4642	<0.0001		<0.0001	<0.0001	
Education									
Primary school or less	15.8 (14.7,16.8)	18.1 (16.9,19.3)	2.4 (1.6,3.2)*^†^*	3.2 (2.9,3.5)	3.8 (3.4,4.2)	0.6 (0.4,0.8)*^†^*	8.4 (7.8,8.9)	9.6 (9.0,10.2)	1.3 (0.9,1.6)*^†^*
Secondary school	18.1 (16.5,19.8)	21.4 (19.9,22.8)	3.2 (2.3,4.2)*^†^*	2.3 (1.9,2.7)	3.4 (2.9,4.0)	1.1 (0.8,1.5)*^†^*	11.1 (10.2,12.0)	13.3 (12.4,14.2)	2.3 (1.7,2.8)*^†^*
High school	22.7 (19.4,26.0)	29.1 (26.3,32.0)	6.4 (4.9,7.9)*^†^*	2.5 (2.0,3.0)	3.7 (2.9,4.5)	1.2 (0.7,1.7)*^†^*	14.3 (12.2,16.4)	18.1 (16.3,19.9)	3.8 (2.8,4.8)*^†^*
College or higher	24.8 (22.7,26.9)	34.9 (32.1,37.7)	10.1 (8.3,11.9)*^†^*	2.7 (2.0,3.5)	3.4 (2.3,4.5)	0.6 (-0.1,1.4)	14.0 (12.9,15.1)	19.0 (17.3,20.6)	4.9 (3.8,6.0)*^†^*
* P* for trend	<0.0001	<0.0001		0.0781	0.4927		<0.0001	<0.0001	
Ethnicity									
Han	19.1 (17.6,20.6)	24.7 (23.2,26.2)	5.6 (4.8,6.3)*^†^*	2.7 (2.5,3.0)	3.6 (3.2,4.0)	0.9 (0.6,1.1)*^†^*	11.0 (10.2,11.8)	14.1 (13.2,15.0)	3.1 (2.7,3.6)*^†^*
Hui	17.3 (12.4,22.2)	18.1 (8.9,27.4)	0.8 (-4.1,5.7)	3.7 (1.2,6.1)	1.7 (0.6,2.8)	-2.0 (-3.4,-0.5)	9.8 (7.4,12.2)	9.2 (4.7,13.7)	-0.6 (-3.0,1.8)
Manchu	17.2 (13.3,21.1)	19.2 (16.3,22.1)	2.1 (-0.6,4.8)	4.1 (2.5,5.8)	7.5 (4.1,10.9)	3.4 (1.3,5.5)	10.3 (8.4,12.3)	13.5 (11.5,15.5)	3.1 (1.6,4.7)*^†^*
Tibetan	27.6 (11.7,43.5)	23.4 (19.2,27.7)	-4.1 (-13.1,4.9)	3.5 (1.6,5.3)	1.8 (0.2,3.5)	-1.6 (-2.8,-0.4)	13.9 (5.8,22.0)	12.4 (9.7,15.1)	-1.5 (-5.7,2.6)
Uighur	5.0 (3.5,6.6)	4.0 (1.3,6.6)	-1.0 (-2.6,0.6)	0.3 (0.0,0.8)	0.2 (0.0,0.4)	-0.2 (-0.5,0.2)	2.8 (1.8,3.8)	2.1 (0.6,3.6)	- 0.7 (-1.7,0.3)
Zhuang	28.4 (24.0,32.9)	32.2 (23.7,40.7)	3.8 (-1.4,8.9)	8.0 (6.0,9.9)	4.5 (2.0,7.0)	-3.5 (-5.3,-1.8)*^†^*	17.1 (14.3,20.0)	17.1 (12.7,21.5)	0.0 (-2.6,2.5)
Others	26.3 (23.5,29.1)	24.7 (20.8,28.5)	-1.6 (-3.8,0.6)	2.9 (1.8,4.0)	3.0 (2.1,3.8)	0.1 (-0.5,0.6)	14.4 (12.9,16.0)	13.3 (11.3,15.4)	-1.1 (-2.1,-0.2)
* P* for difference	<0.0001	<0.0001		<0.0001	<0.0001		<0.0001	<0.0001	
Income per capita (CNY)									
Q1 (¥<6000)	15.1 (13.8,16.3)	17.4 (15.6,19.1)	2.3 (1.2,3.4)*^†^*	2.7 (2.3,3.2)	2.9 (2.4,3.5)	0.2 (- 0.2,0.6)	9.0 (8.3,9.6)	10.2 (9.3,11.2)	1.3 (0.7,1.9)*^†^*
Q2 (¥6000-11999)	16.4 (15.0,17.7)	20.5 (18.2,22.8)	4.1 (2.7,5.6)*^†^*	2.7 (2.3,3.2)	3.2 (2.5,3.8)	0.5 (0.1,0.9)	9.5 (8.8,10.3)	11.9 (10.6,13.2)	2.4 (1.6,3.2)*^†^*
Q3 (¥12000-23999)	19.5 (18.1,20.9)	25.9 (23.7,28.0)	6.4 (5.1,7.6)*^†^*	2.6 (2.3,3.0)	4.0 (3.3,4.7)	1.4 (0.9,1.8)*^†^*	11.2 (10.4,12.0)	14.8 (13.5,16.1)	3.7 (2.9,4.4)*^†^*
Q4 (≥¥24000)	24.6 (21.9,27.2)	29.6 (27.1,32.1)	5.0 (4.0,6.0)*^†^*	2.8 (2.5,3.1)	4.1 (3.3,4.8)	1.3 (0.9,1.7)*^†^*	13.9 (12.4,15.4)	16.9 (15.3,18.5)	3.0 (2.5,3.6)*^†^*
Refused/Don’t know *^‡^*	19.6 (17.4,21.8)	25.1 (22.5,27.6)	5.4 (3.7,7.2)*^†^*	3.0 (2.5,3.6)	3.5 (2.9,4.1)	0.4 (0.0,0.8)	11.1 (10.0,12.2)	13.9 (12.5,15.3)	2.8 (1.9,3.7)*^†^*
* P* for trend	<0.0001	<0.0001		0.9119	0.0060		<0.0001	<0.0001	

^*^Data are presented incorporating sample weights and adjusted for clusters and strata of the complex sample design of CCDRFS. ^†^P values for changes from 2015-16 to 2018-19 are <0.05. ^‡^Participants who answered “I don’t know or I don’t want to tell you” are not included in the trend test. ^§^P values for difference between men and women are <0.05. 95% CI=95% confidence interval. NA, data not available. ¥ 100= £11/€13/$15.5.

**Table 3:** Prevalence of hyperuricemia among adults in mainland China by risk factors and major chronic diseases, 2015-16 and 2018-19*.

Characteristics	Prevalence, 95% CI								
	Men			Women			Overall		
	2015-16	2018-19	Changes of prevalence, 2018-19 vs 2015-16	2015-16	2018-19	Changes of prevalence, 2018-19 vs 2015-16	2015-16	2018-19	Changes of Prevalence, 2018-19 vs 2015-16
Current smoking									
Yes	18.4(17.2,19.7)	22.7(21.3,24.1)	4.3(3.5,5.0)*^†^*	3.7(2.7,4.7)	3.7(2.7,4.7)	0.0(-0.7,1.0)	17.9(16.7,19.0)	21.9(20.6,23.3)	4.1(3.4,4.8)*^†^*
No	20.3(18.5,22.1)	26.2(24.2,28.1)	5.9(4.9,6.8)*^†^*	2.8(2.5,3.0)	3.6(3.2,4.0)	0.9(0.6,0.0)*^†^*	8.5(7.9,9.2)	13.0(12.2,13.8)	2.7(2.3,3.1)*^†^*
* P* for difference	0.0047	<0.0001		0.0436	0.8691		<0.0001	<0.0001	
Heavy drinking									
Yes	22.5(19.5,25.6)	26.2(24.2,28.2)	3.7(2.0,5.4)*^†^*	3.4(2.5,4.2)	5.5(3.5,7.5)	2.2(1.0,0.1)	18.5(16.2,20.7)	24.8(23.0,26.6)	6.3(5.0,7.7)*^†^*
No	18.5(17.4,19.7)	24.1(22.7,25.6)	5.6(4.9,6.3)*^†^*	2.7(2.5,3.0)	3.6(3.2,4.0)	0.9(0.6,0.0)*^†^*	10.0(9.4,10.6)	13.0(12.2,13.8)	3.0(2.6,3.4)*^†^*
*P* for difference	<0.0001	<0.0001		0.1588	0.0309		<0.0001	<0.0001	
Beer drinking									
Never	17.9(17.0,18.9)	23.0(21.6,24.4)	5.1(4.3,5.9)*^†^*	2.8(2.6,3.1)	3.7(3.3,4.1)	0.9(0.6,1.1)*^†^*	9.0(8.5,9.4)	11.9(11.1,12.6)	2.9(2.4,3.3)*^†^*
At least once/year	20.5(17.9,23.1)	26.0(23.5,28.5)	5.5(4.2,6.9)*^†^*	2.0(1.5,2.6)	2.8(2.0,3.5)	0.7(0.2,1.2)	15.6(13.6,17.6)	19.3(17.4,21.1)	3.7(2.7,4.6)*^†^*
At least once/week	23.1(19.9,26.2)	29.2(26.7,31.8)	6.2(4.4,7.9)*^†^*	4.7(2.1,7.2)	3.7(2.1,5.3)	-1.0(-2.6,0.6)	21.5(18.5,24.4)	27.1(24.8,29.4)	5.6(4.0,7.3)*^†^*
* P* for trend	<0.0001	<0.0001		0.9621	0.1290		<0.0001	<0.0001	
Fruit & vegetable intake <400g/d									
Yes	18.8(17.6,20.0)	25.1(22.8,27.4)	6.3(5.2,7.4)*^†^*	2.9(2.6,3.3)	3.3(3.0,3.7)	0.4(0.2,0.7)	11.0(10.2,11.7)	14.4(13.1,15.7)	3.4(2.8,4.1)*^†^*
No	19.9(18.1,21.7)	23.9(22.6,25.1)	4.0(3.1,4.9)*^†^*	2.6(2.4,2.9)	3.8(3.3,4.3)	1.2(0.9,1.5)*^†^*	11.2(10.3,12.1)	13.6(12.9,14.4)	2.4(2.0,2.9)*^†^*
* P* for difference	0.1026	0.33		0.1077	0.1112		0.5070	0.2584	
Red meat intake >100g/d									
Yes	23.2(21.7,24.7)	29.2(27.3,31.1)	6.0(5.2,6.8)*^†^*	2.9(2.6,3.3)	4.7(3.8,5.5)	1.7(1.1,2.2)*^†^*	15.3(14.4,16.2)	19.0(17.6,20.5)	3.7(3.1,4.4)*^†^*
No	17.3(16.0,18.7)	19.8(18.3,21.3)	2.5(1.5,3.5)*^†^*	2.6(2.4,2.9)	3.0(2.7,3.4)	0.3(0.1,0.5)	10.7(9.9,11.5)	13.3(12.4,14.1)	0.9(0.4,1.4)
*P* for difference	<0.0001	<0.0001		0.2911	<0.0001		<0.0001	<0.0001	
Physical inactivity (<150min/w)									
Yes	20.0(18.5,21.6)	26.1(24.0,28.2)	6.1(4.9,7.3)*^†^*	3.0(2.5,3.6)	4.6(3.8,5.3)	0.7(0.4,1.0)*^†^*	12.5(11.5,13.4)	16.4(15.0,17.7)	3.9(3.1,4.7)*^†^*
No	19.1(17.5,20.8)	23.9(22.4,25.3)	4.8(4.0,5.5)*^†^*	2.7(2.5,2.9)	3.4(3.0,3.8)	1.3(0.9,1.8)*^†^*	10.7(9.9,11.5)	13.3(12.4,14.1)	2.6(2.2,3.0)*^†^*
* P* for difference	0.3864	0.0349		0.0372	0.0021		0.0018	<0.0001	
BMI group (kg/m^2^)									
<18.5	9.8(7.2,12.3)	17.5(11.1,23.8)	7.7(3.5,11.9)	1.4(0.9,1.8)	0.8(0.4,1.2)	-0.6(-0.9,-0.2)	5.2(4.1,6.4)	8.4(5.0,11.8)	3.2(1.1,5.3)
18.5-24.9	14.6(12.8,16.4)	17.6(16.3,18.9)	3.0(2.1,3.8)*^†^*	1.9(1.6,2.1)	2.4(2.1,2.7)	0.5(0.3,0.7)*^†^*	8.0(7.1,9.0)	9.5(8.8,10.2)	1.4(1.0,1.8)*^†^*
25.0-29.9	25.4(23.7,27.2)	29.8(28.0,31.5)	4.3(3.3,5.4)*^†^*	3.6(3.1,4.0)	5.0(4.3,5.8)	1.5(1.0,1.9)*^†^*	15.4(14.3,16.4)	18.5(17.4,19.7)	3.2(2.5,3.8)*^†^*
≥30.0	35.8(33.3,38.2)	46.9(43.0,50.7)	11.1(8.7,13.5)*^†^*	9.1(7.8,10.4)	10.0(8.1,11.8)	0.9(-0.3,2.1)	22.6(21.3,24.0)	30.1(27.4,32.9)	7.5(5.9,9.1)*^†^*
* P* for trend	<0.0001	<0.0001		<0.0001	<0.0001		<0.0001	<0.0001	
Central obesity									
Yes	27.4(25.6,29.1)	33.4(31.5,35.3)	6.0(4.9,7.2)*^†^*	5.0(4.6,5.5)	6.1(5.4,6.9)	1.1(0.7,1.6)*^†^*	16.5(15.5,17.5)	20.5(19.2,21.8)	4.0(3.3,4.7)*^†^*
No	15.9(14.3,17.4)	19.1(17.6,20.7)	3.3(2.5,4.0)*^†^*	1.9(1.6,2.1)	2.4(2.0,2.7)	0.5(0.3,0.7)*^†^*	8.8(8.0,9.6)	10.5(9.6,11.3)	1.6(1.2,2.0)*^†^*
* P* for difference	<0.0001	<0.0001		<0.0001	<0.0001		<0.0001	<0.0001	
Hypertension									
Yes	22.1(20.7,23.5)	27.0(25.5,28.5)	4.9(3.9,5.8)*^†^*	4.8(4.3,5.3)	6.3(5.5,7.0)	1.5(1.0,1.9)*^†^*	14.2(13.3,15.0)	17.8(16.8,18.8)	3.6(3.0,4.3)*^†^*
No	18.0(16.4,19.7)	23.3(21.7,24.9)	5.3(4.5,6.1)*^†^*	2.0(1.8,2.3)	2.8(2.4,3.2)	0.7(0.5,1.0)*^†^*	10.6(9.9,11.4)	12.5(11.6,13.5)	2.7(2.3,3.1)*^†^*
*P* for difference	<0.0001	<0.0001		<0.0001	<0.0001		<0.0001	<0.0001	
Diabetes status									
Normal	NA	24.1(22.5,25.7)	NA	NA	2.6(2.3,3.0)	NA	NA	12.9(12.0,13.8)	NA
Prediabetes	NA	26.9(24.9,29.0)	NA	NA	5.6(4.7,6.5)	NA	NA	17.2(15.8,18.5)	NA
Diabetes without treatment	NA	26.0(23.6,28.4)	NA	NA	7.3(6.2,8.4)	NA	NA	18.2(16.6,19.7)	NA
Diabetes treated	NA	16.8(14.3,19.4)	NA	NA	6.9(5.9,8.0)	NA	NA	11.7(10.1,13.2)	NA
*P* for difference		<0.0001			<0.0001			<0.0001	
Low eGFR									
Yes	NA	52.6(49.3,56.0)	NA	NA	25.3(22.6,28.0)	NA	NA	38.0(35.7,40.3)	NA
No	NA	23.8(22.4,25.3)	NA	NA	3.1(2.7,3.4)	NA	NA	13.4(12.6,14.3)	NA
*P* for difference		<0.0001			<0.0001			<0.0001	
Albuminuria									
Yes	NA	31.4(28.7,34.1)	NA	NA	6.8(5.9,7.7)	NA	NA	18.5(16.8,20.2)	NA
No	NA	23.9(22.6,25.3)	NA	NA	3.4(3.0,3.8)	NA	NA	13.8(12.9,14.6)	NA
*P* for difference		<0.0001			<0.0001			<0.0001	
High TC									
Yes	30.3(27.3,33.2)	36.2(32.4,39.9)	5.9(4.1,7.7)*^†^*	6.5(5.4,7.6)	8.9(7.1,10.7)	2.4(1.2,3.5)*^†^*	17.9(16.5,19.2)	22.7(20.4,25.1)	4.9(3.7,6.0)*^†^*
No	18.7(17.3,20.0)	23.3(22.0,24.7)	4.7(3.9,5.4)*^†^*	2.5(2.3,2.8)	3.1(2.8,3.5)	0.6(0.4,0.8)*^†^*	10.6(9.9,11.4)	13.2(12.4,13.9)	2.5(2.1,2.9)*^†^*
*P* for difference	<0.0001	<0.0001		<0.0001	<0.0001		<0.0001	<0.0001	
High TG									
Yes	34.1(32.3,35.9)	40.7(38.4,43.1)	6.6(5.2,8.0)*^†^*	8.5(7.4,9.6)	8.8(7.7,9.8)	0.3(-0.5,1.1)	24.6(23.4,25.8)	29.3(27.4,31.1)	4.6(3.6,5.7)*^†^*
No	15.9(14.3,17.5)	19.4(18.1,20.7)	3.5(2.7,4.2)*^†^*	2.1(1.9,2.2)	2.8(2.5,3.2)	0.8(0.5,1.0)*^†^*	8.7(7.9,9.5)	10.5(9.8,11.3)	1.8(1.5,2.2)*^†^*
*P* for difference	<0.0001	<0.0001		<0.0001	<0.0001		<0.0001	<0.0001	

^*^Data are presented incorporating sample weights and adjusted for clusters and strata of the complex sample design of CCDRFS. ^†^P values for changes from 2015-16 to 2018-19 are <0.05. 95% CI, 95% confidence interval. NA, data not available; BMI, body mass index; TC, total cholesterol; TG, triglycerides; eGFR, estimated glomerular filtration rate.

Between 2015–16 and 2018–19, the HUA prevalence had increased by 2.9% (95% CI, 2.5–3.3%, *P*<0.05). The increase was higher in men, younger people (18-29 and 30-39 years old), the best educated (those who finished college or above), urban adults, and those living in Eastern China (all *P <*0.05). Compared to 2015–16, the HUA prevalence increased in 2018–19 in the Han (*P <*0.05) and Manchu (*P <*0.05) groups, while no significant changes were observed in the other ethnic groups (**Table 2**). Furthermore, the prevalence increased more rapidly between the two periods in adults with health-related risk factors, including smoking, heavy drinking, high red meat intake, physical inactivity, BMI ≥25 kg/m^2^, central obesity, high TC and TG levels (all *P <*0.05, **Table 3**). The estimated HUA prevalence in women increased from 9.6 to 11.5% between 2015–16 and 2018–19, when HUA was defined as SUA >360 µmol/L (**Supplementary Table 3**).

We further estimated the mean levels of SUA. From 2015–16 to 2018–19, the mean levels of SUA among Chinese adults increased from 310.0 µmol/L (95% CI 306.6– 313.4 µmol/L) to 320.8 µmol/L (95% CI 317.8–323.9 µmol/L, P<0.0001), with a higher increase observed in men (**Supplementary Table 2**). It reached 387.3 µmol/L (95% CI 379.7–394.9 µmol/L) in men aged 18–29 years in 2018–19 (**Supplementary Table 4**). Furthermore, the percentage with severely high SUA levels increased notably; 15.3% had SUA values >540 µmol/L and 5.3% had SUA values >600 µmol/L. Severely high SUA levels were more common in the urban residences, and the adults with younger age or higher education (**Supplementary Table 5**).

**Figure 3:**
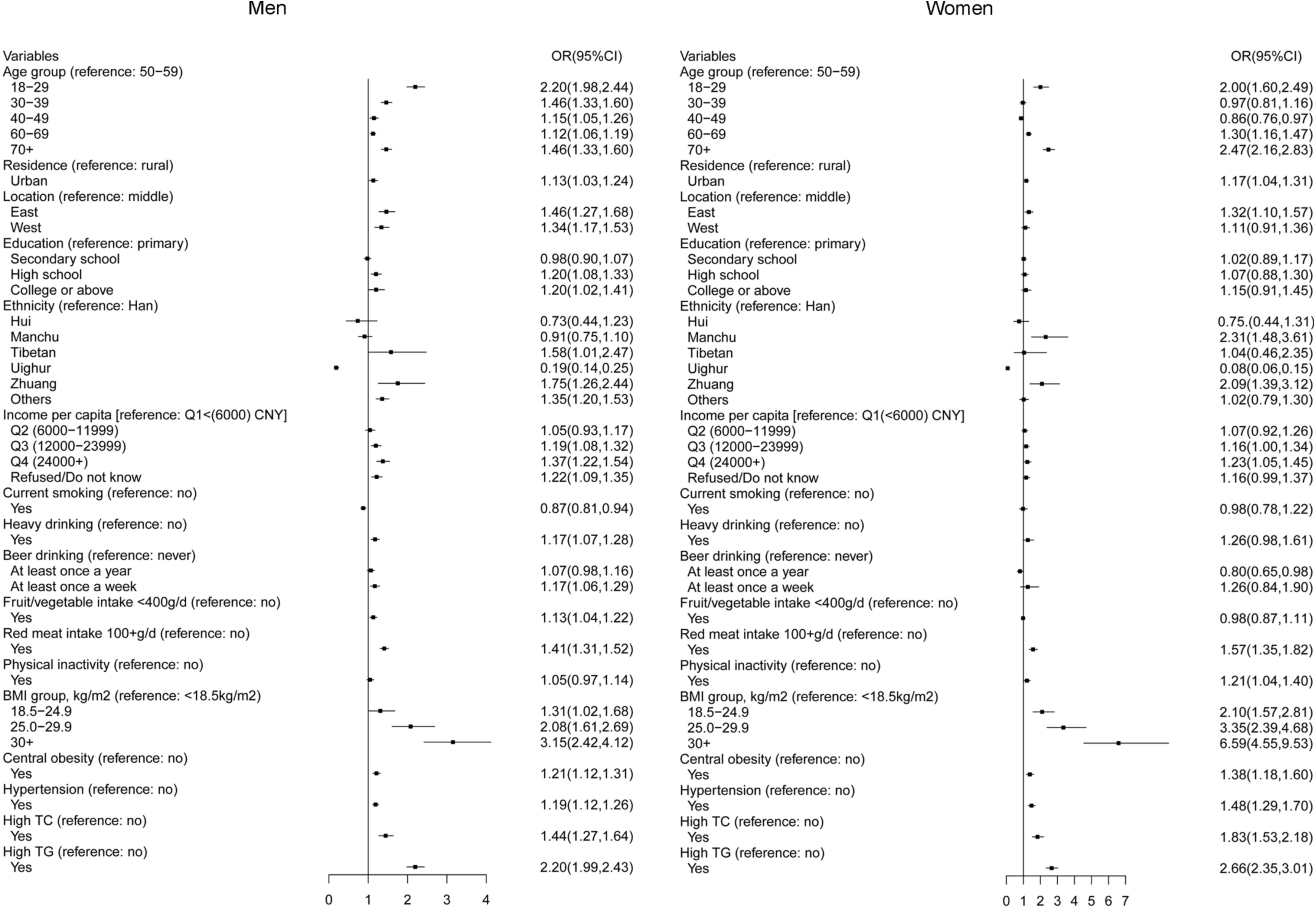
Risk factors associated with hyperuricemia among adults in mainland China.

The risk factors for HUA were evaluated using data combined from the two surveys (**Figure 3**). Factors that were significantly associated with HUA include age between 18 and 29 years or above 70 years, urban residency, location in East, Zhuang descent, high income, red meat intake >100 g/d, physical inactivity, high BMI, central obesity, hypertension, and high TC and TG levels. In addition, Tibetan descent, age 30–39 years, heavy alcohol/beer drinking at least once a week, and high educational qualification were risk factors of HUA in men. Smoking was inversely correlated with HUA in men. Based on the 2018–19 data, prediabetes, diabetes, low eGFR, and albuminuria were notable risk factors associated with HUA.

## Discussion

This nationwide study estimated that approximately 14.0% of Chinese adults (18 years or older) had HUA in 2018–19, among them 15.2% had an SUA value >540 µmol/L and 5.3% had an SUA value >600 µmol/L. HUA prevalence had significantly increased in three years from 11.1% in 2015–16 to 14.0% in 2018–19. In the national survey in 2009–10, the overall HUA prevalence in Chinese adults was 8.4% (9). The sustained increase in the prevalence of HUA in the past decade indicates that HUA has reached warning levels in the general Chinese population.

The HUA prevalence in China is similar to that in developed countries. A national survey in Japan reported an HUA prevalence of 13.4% in 2016–17 (21), while national research in the United States (US) reported HUA prevalences of 14.6% in 2015–16 and 15.9% in 2007–08, suggesting that the HUA prevalence in the US was stable over the past decade (22). However, the HUA prevalence is steadily growing in China, attributed to its booming economy and large-scale urbanization (23). Economic development has brought about lifestyle changes that have increased the prevalence of metabolic diseases, such as HUA, obesity, diabetes, and hypertension (24). Moreover, increased red meat intake, physical inactivity, higher BMI, and central obesity were observed in 2018–19 compared to that in 2015–16.

HUA appears to be more common in men. A prevalence of 24.7% in men and 5.2% in women was reported in the US in 2015–16 (22). Our values were similar to these, with a prevalence of 24.4% in men and 3.6% in women in 2018–19 in China. The lower HUA prevalence and SUA levels in women may be hormonal, attributed to the effects of estrogen, or their lifestyles (25, 26). HUA prevalence in women in China was reported to be 7.0% in a national survey in 2009–10 with HUA defined as SUA>360 µmol/L. This percentage rose to 11.5% in 2018–19, when HUA was defined to be SUA >360 µmol/L in our survey; 7.9% of Chinese women had SUA levels between 360 and 420 µmol/L. However, it is inappropriate to classify women with SUA levels of 360–420 µmol/L as positive for HUA because there is lacking of definited evidence that SUA levels >360 µmol/L in women are beyond the saturation level in blood and cause possible pathological injury. In this study, we have defined HUA with an SUA level >420 µmol/L, regardless of sex (27, 28), although research has shown that the SUA threshold for all-cause mortality was 320 µmol/L in men and 280 µmol/L in women in Italian cohorts (29). Above all, The prevalence of HUA in Chinese women has been rising steadily in the past decade, and the female population with SUA levels of 360–420 µmol/L may be at a high risk of developing HUA.

The risk factors for HUA were evaluated in this study. Age (18–29 or >70 years), urban culture, geographical location (settlers in the East), high education, Zhuang descent, heavy drinking or frequent beer drinking, high red meat intake, physical inactivity, high BMI or central obesity, hypertension, hyperlipidemia, prediabetes, diabetes, and low eGFR were risk factors for HUA. We observed a significantly high prevalence amongst young people aged 18-29 years old in China, which may be a result of unhealthy lifestyles, including high-stress levels at work, the habit of eating out, physical inactivity, and high-fructose intake (7, 26, 30–32). Recent research has shown that HUA occurs earlier than other metabolic disorders, including hypertension, hypertriglyceridemia, and diabetes mellitus, suggesting that HUA may play an upstream role in cardio-metabolic disease development (33). HUA has been confirmed to be an independent risk factor for metabolic disorders, and in our research the metabolic disorders, including obesity, hypertension, hyperlipidemia, and diabetes, have shown associations with HUA.

Eastern China has a relatively higher prevalence of metabolic diseases than the rest of the country (34, 35). A regional survey of the eastern Chinese population from January 2014 to December 2015 sh2owed an HUA prevalence of 11.3% (11). Our results showed that the prevalence, which was 12.2% in 2015–16, ascended to 17.1% in 2018–19. It may be attributed to the developed economy (36) and abundant seafood consumption in the coastal areas (10).

We observed notable differences in HUA prevalence among different ethnicities in China. The prevalence was 14.1% in the Han, as high as 17.1% in the Zhuang, as low as 2.1% in the Uighur in 2018–19. The mean SUA level was also strikingly lower in the Uighur than in the other ethnic group. These ethnic variations of HUA prevalence may be associated with their different lifestyles. A recent survey in Xinjiang reported a similarly low HUA prevalence in the Uighur community (37), and correlated it with the low alcohol intake in Uighur. However, apart from lifestyles, genetic backgrounds may potentially influence the HUA prevalence among the different ethnic groups.

To our knowledge, the CCDRFS is the only surveillance that provide both national and preovincial representative information on chronic diseases and risk factors, including uric acid level in China. Standardized survey instruments, consistent sampling method, high participants acceptance rate, and quality control protocol guarantee the reliablitly and comparability of the results over the years. Espeicially, all blood samples were tested for uric acid by standard option process in the central laboratory, which provide us high quality data. Nevertheless, two limitations should be considered when interpreting the study results. First, women and rural people were overly sampled in this study. To control this, all results were weighted by the Chinese population distribution. Second, it was a cross-sectional survey, which hampered the study’s ability to determine the causal relationship between risk factors and hyperuricemia development.

In conclusion, from the two large-scale national surveys, we observed an HUA prevalence of 14.0% in the Chinese population (approximately 19.19 million individuals) in 2018–19. The prevalence has been constantly increasing, with the younger generation being affected more. These findings indicate the importance of HUA as a public health problem in China.

## Data Availability Statement

The datasets presented in this article are not readily available because individual participant data in our study will not be made available publicly. Requests to access the datasets should be directed to LW, jianceshi@ncncd.chinacdc.cn.

## Ethics Statement

The studies involving human participants were reviewed and approved by the ethical review committee of the Chinese Center for Disease Control and Prevention (China CDC) and the ethical review committee of the National Center for Chronic and Noncommunicable Disease Control and Prevention, China CDC. The patients/participants provided their written informed consent to participate in this study.

## Author Contributions

LMW, MGZ, JW, and MZ had full access to all the data in the study and take responsibility for the integrity of the data and accuracy of the data analysis. MGZ, LMW, HZ, MZ, and XXZ conceived and designed the study. All authors acquired, or interpreted the data. MZ and XXZ drafted the manuscript. LMW, HZ, and MGZ critically revised the manuscript for intellectual content. MZ statistically analyzed the data. ZZ and XZ verified the underlying data. MZ and LMW obtained funding. JW, MGZ, and LHW provided administrative, technical, or material support. MGZ, HZ, and LMW supervised the study. All authors contributed to the article and approved the submitted version.

## Funding

The surveillance was funded by the Chinese Central Government (Key Project of Public Health Program). This study was supported by the National Key R&D Program of China (grant numbers 2018YFC1311702 and 2018YFC1311706).

## Conflict of Interest

The authors declare that the research was conducted in the absence of any commercial or financial relationships that could be construed as a potential conflict of interest.

## Publisher’s Note

All claims expressed in this article are solely those of the authors and do not necessarily represent those of their affiliated organizations, or those of the publisher, the editors and the reviewers. Any product that may be evaluated in this article, or claim that may be made by its manufacturer, is not guaranteed or endorsed by the publisher.
