# Supplementary material for: Prevalence of Hyperuricemia Among Chinese Adults: Findings From Two Nationally Representative Cross-Sectional Surveys in 2015–16 and 2018–19
Source: Front Immunol. 2022 Feb 7;12:791983. doi: 10.3389/fimmu.2021.791983 (PMC8858821; doi:10.3389/fimmu.2021.791983)
Supplement: Supplementary file 1 [file DataSheet_1.docx]

**Supplementary materials**

**Prevalence of Hyperuricemia among Chinese Adults: Findings from Two Nationally Representative Cross-Sectional Surveys in 2015–16 and 2018–19,** by Mei Zhang, et al.

**Table of contents**

[Supplementary 1. Sample design of CCDRFS 2015 and CCDRFS 2018 3](#_Toc80016033)

[Supplementary Figure 1: Map of China Chronic Disease and Risk Factor Surveillance Counties or Districts 7](#_Toc80016034)

[Supplementary Table 1: Laboratory testing methods in CCDRFS 2015 and CCDRFS 2018 8](#_Toc80016035)

[Supplementary Table 2: Means (µmol/L, 95%CI) of blood uric acid among Chinese adults by sociographic characteristics, 2015-16 and 2018-19 9](#_Toc80016036)

[Supplementary Table 3: Prevalence of hyperuricemia diagnosed with different criteria in Chinese women aged 18 years and above, 2015-16 and 2018-19 10](#_Toc80016037)

[Supplementary Table 4: Means (µmol/L, 95%CI) of blood uric acid among men and women by sociographic characteristics, 2015-16 and 2018-19 11](#_Toc80016038)

[Supplementary Table 5: Proportions of hyperuricemia by levels among Chinese adults by sociodemographic characteristics, 2015-16 and 2018-19 12](#_Toc80016039)

# Supplementary 1. Sample design of CCDRFS 2015 and CCDRFS 2018

**Overview**

The sample of CCDRFS was obtained from China’s national Disease Surveillance Point (DSP) system.^1^ One DSP unit covers a rural county or an urban district. The DSPs were established during early1980s, using multi-stage stratified sampling with probability proportional to size (PPS) to ensure representativeness of the national population of mainland China. Following further expansion and sampling enhancement, from the 2013 survey the selected DSPs were also representative at provincial level. CCDRFS is planned and administrated by the National Center for Chronic and Non-communicable Disease Control and Prevention (NCNCD) of the Chinese Center for Disease Control and Prevention (CDC).

**Disease Surveillance Points (DSPs) system**

The DSPs system was piloted in 1978 and fully established in early 1980s covering initially 71 sites, expanding to 145 in 1989 and 161 DSPs in 2004 (covering a population of 73 million), in order to accommodate the societal and economic development during this period. For each expansion, the population characteristics of the selected DSPs were compared to the census population to ensure its nationally representativeness.^2,3^ In 2013, the Chinese government combined the DSPs system with the national vital registration system to form an integrated national mortality surveillance system, which increased the DSPs areas from 161 to 605. The new DSPs system currently covers 324 million Chinese adults (24% of all Chinese population) in the mainland of China.

**Sampling procedure**

Embedded within the DSP system, the CCDRFS 2015 and CCDRFS 2018 used stratified multi-stage cluster sampling to generate a nationally representative sample for each survey.

In the first stage, 298 were selected from all 605 DSPs as primary sampling units (PSUs) using stratified sampling to generate a sample representative of both the national and provincial population in the 31 provinces of mainland China. The sampling of DSPs was done centrally by the NCNCD.

Then, within each selected DSP, the following steps were followed:

1. Townships (rural) or subdistricts (urban) were selected with probability proportional to size (PPS) sampling in each selected DSP.
2. Within the selected townships and subdistricts, villages (rural) or residential areas (urban) were selected with PPS sampling.
3. Each selected village or residential area was divided into groups of about 60 households, based on existing villager/resident groups in the village or residential area. One group was selected with simple random sampling.
4. Within each selected household, all eligible adults in the household were invited.

Eligibility criteria include:

- 1. Aged 18 years or older;
  2. Having lived in the address for more than 6 months in the past 12 months;
  3. Not pregnant;
  4. Not having a serious health condition or illness that prevents from participating, including intellectual disability or language disorder.

1. In a household, if more than half of the family’s eligible members refused to participate or were not reachable, a replacement household with similar sex and age structure was selected. In each round, <10% households were replaced.

The sampling within each DSP was performed by the provincial CDCs and approved by the NCNCD.

**Sample size calculation**

Sample size of each round of CCDRFS per stratum was calculated using N =

where *u* = 1.96 (corresponding to 95% confidence level), *deff* is design efficiency (3), *r* is relative error (20%), and *d* = *r* × *p*. *p* is the prevalence of factor studied for calculation. For tow surveys, diabetes prevalence (9.7%) in 2010 and 10.4% in 2013 was used, respectively. The non-response rates (15% in 2015 and 10% in 2018) were also included in the calculation. The table below shows the calculated target sample sizes in each survey. Since the previous reported hyperuricemia prevalence was higher than the prevalence of diabetes in each survey year, the calculated sample size could satisfy the accurate estimation of hyperuricemia rate.

| Survey | 2015 | 2018 |
| --- | --- | --- |
| Target sample size | 184,773 | 181,059 |

**Weights calculation**

Across CCDRFS 2015 and CCDRFS 2018 surveys, we developed sample weights to account for multi-stage sampling design, post-stratification, and non-response within household as all members were selected. For an individual in the sample, his/her sample weights were developed as follows.

1. Base weights for multi-stage design (Wdesign)

Wdesign = Wd1 × Wd2 × Wd3 × Wd4 × Wd5

- Wd1 is the total number of counties (rural) or districts (urban) in the stratum divided by the number of selected DSPs (PSU) in the stratum where the individual was from;
- Wd2 is the total number of townships (rural) or subdistricts (urban) in the PSU where the individual belonged divided by the number of selected townships or subdistricts;
- Wd3 is the total number of villages (rural) or residential areas (urban) in the township or subdistrict where the individual was from divided by the number of selected villages or residential areas;
- Wd4 is the total number of groups in the village or residential area where the individual was from;
- Wd5 is the total number of households in the group where the individual belonged divided by the number of selected households in the group where the individual was from.

1. Non-response weights (Wnr) in the 2015 and 2018 surveys

Wnr = the number of eligible adults in the household where the individual was from divided by the number of participating adults in the household.

1. Post-stratification weights (Wps)

Stratifications included: province (31 levels), urban or rural (2 levels), gender (2 levels), age group (10 levels: 18-24, 25-29, 30-34, 35-39, 40-44, 45-49, 50-54, 55-59, 60-64, 65-69). The 2010 census population was also stratified in the same way. In k^th^ stratum, the post-stratification weights (Wps,k) are:

$$Wps,k= \frac{Population in the k^{\mathrm{th}} stratum of the 2010 census population}{Sum of Wdesign\times Wnr for all individuals in the k^{\mathrm{th}}\mathrm{stratum}}$$

**References**

1. Yang G, Hu J, Rao KQ, Ma J, Rao C, Lopez AD. Mortality registration and surveillance in China: History, current situation and challenges. *Popul Health Metr* 2005; **3**: 3.

2. Yang G. [Selection of DSP points in second stage and their presentation]. *Zhonghua Liu Xing Bing Xue Za Zhi* 1992; **13**: 197-201.

3. Zhou MG, Jiang Y, Huang ZJ, Wu F. Adjustment and representativeness evaluation of national disease surveillance points system. *Dis Surveill* 2010; **25**: 239-44.


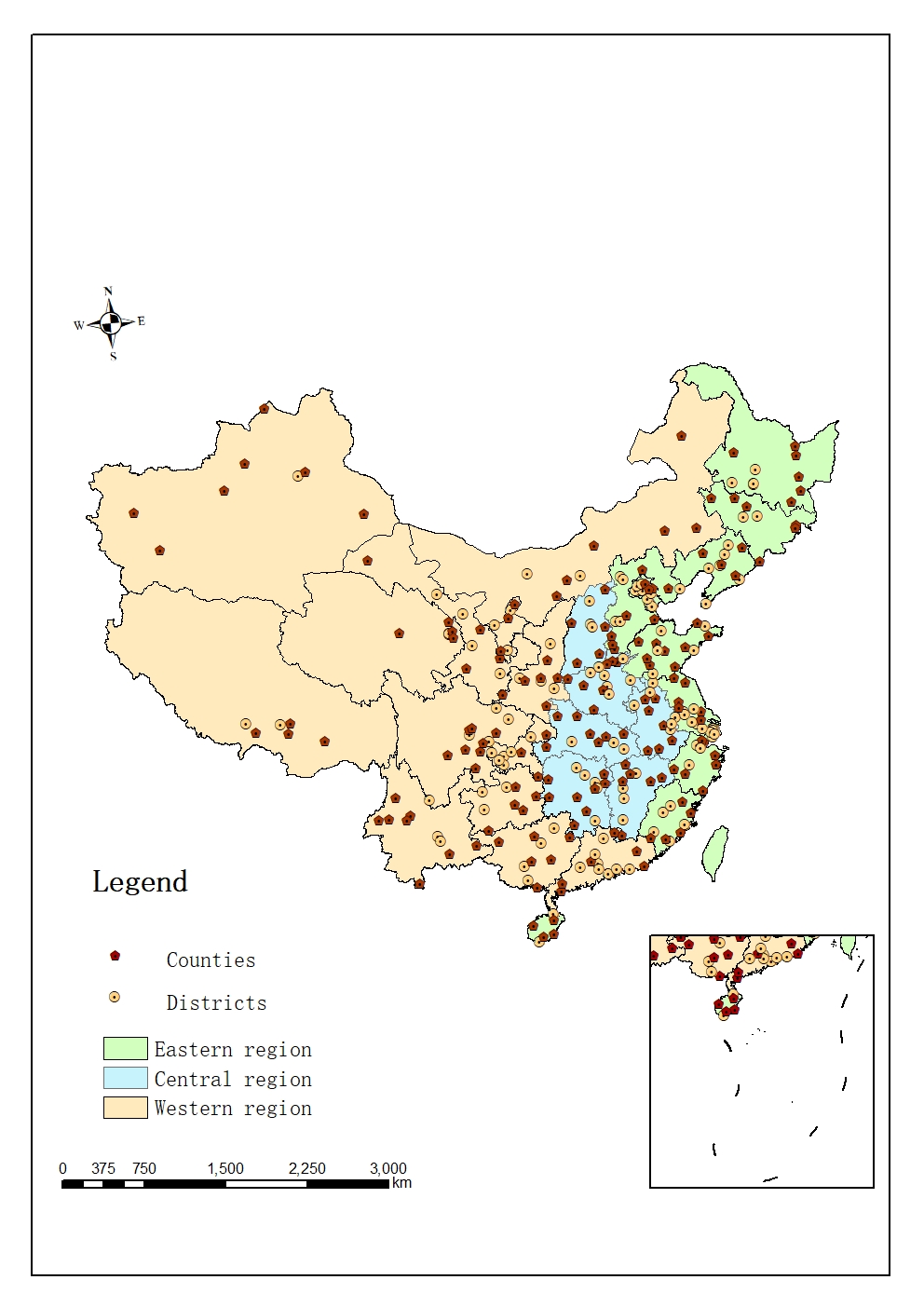


# Supplementary Figure 1: Map of China Chronic Disease and Risk Factor Surveillance Counties or Districts

# Supplementary Table 1: Laboratory testing methods in CCDRFS 2015 and CCDRFS 2018

| **Sample type** | **Indicators** | **CCDRFS 2015** | | **CCDRFS 2018** | |
| --- | --- | --- | --- | --- | --- |
|  |  | **Methods** | **Equipment** | **Methods** | **Equipment** |
| Serum | Blood uric acid | Uricase peroxidase method | Hitachi 7600 automated clinical chemistry analyzer (Hitachi, Ltd· Japan) | Uricase peroxidase method | Roche cobas 8000 modular analyzer series (Roche, Ltd· Switzerland) |
|  | Total cholesterol | Cholesterol oxidase amino antipyrine method |  | Cholesterol oxidase amino antipyrine method |  |
|  | Triglyceride | Phosphoglycerol oxidase 4-chloric acid method |  | Phosphoglycerol oxidase 4-chloric acid method |  |
|  | Blood creatinine | NA |  | Enzyme-coupled sarcosine oxidase |  |
| Plasma | Plasma glucose | NA |  | Hexokinase method | Automated clinical chemistry analyzers in local CDCs |
| Urine | Urine microalbumin | NA |  | Immunoturbidimetry | Roche cobas 8000 modular analyzer series (Roche, Ltd· Switzerland) |
|  | Urine creatinine | NA |  | Enzyme-coupled sarcosine oxidase |  |

NA=not applicable.

# Supplementary Table 2: Means (µmol/L, 95%CI) of blood uric acid among Chinese adults by sociographic characteristics, 2015-16 and 2018-19

|  | **Mean (µmol/L, 95%CI) of blood uric acid** | | **Changes of means, mean (µmol/L, 95%CI) 2018-19 vs 2015-16** | **p value for difference** |
| --- | --- | --- | --- | --- |
|  | **2015-16** | **2018-19** |  |  |
| **Total** | 310·0(306·6-313·4) | 320·8(317·8-323·9) | 10·8(9·3-12·3) | <0·0001 |
| **Gender** |  |  |  |  |
| Men | 351·4(347·3-355·5) | 365·5(361·6-369·5) | 14·2(12·2-16·1) | <0·0001 |
| Women | 268·4(265·8-271·0) | 276·5(274·3-278·8) | 8·2(6·8-9·5) | <0·0001 |
| **Age group (years)** |  |  |  |  |
| 18-29 | 319·2(314·0-324·3) | 334·9(329·2-340·5) | 15·7(13·1-18·2) | <0·0001 |
| 30-39 | 309·2(305·9-312·6) | 323·0(319·2-326·9) | 13·8(12·0-15·6) | <0·0001 |
| 40-49 | 303·5(299·1-308·0) | 310·3(307·6-313·0) | 6·8(4·3-9·2) | 0·0062 |
| 50-59 | 305·6(302·7-308·5) | 313·3(310·4-316·3) | 7·8(5·9-9·7) | <0·0001 |
| 60-69 | 307·0(304·1-309·8) | 313·7(310·8-316·5) | 6·7(4·8-8·6) | 0·0006 |
| ≥70 | 315·5(310·6-320·4) | 324·4(320·9-327·9) | 8·9(5·8-12·0) | 0·0044 |
| **Residence** |  |  |  |  |
| Urban | 317·3(312·4-322·3) | 330·0(326·0-334·1) | 12·7(10·4-15·0) | <0·0001 |
| Rural | 302·4(299·8-305·0) | 311·0(307·8-314·1) | 8·6(6·9-10·3) | <0·0001 |
| **Education** |  |  |  |  |
| Primary school or less | 297·9(295·0-300·8) | 303·2(300·4-306·0) | 5·3(3·6-7·0) | 0·0022 |
| Secondary school | 310·6(307·5-313·7) | 319·8(316·8-322·8) | 9·2(7·4-11·0) | <0·0001 |
| High school | 324·4(317·2-331·5) | 337·3(332·1-342·6) | 13·0(9·9-16·0) | <0·0001 |
| College or higer | 321·7(318·5-324·9) | 338·3(332·7-343·9) | 16·5(13·7-19·4) | <0·0001 |
| **Ethnic** |  |  |  |  |
| Han | 310·1(306·4-313·7) | 321·6(318·3-324·9) | 11·5(9·9-13·1) | <0·0001 |
| Hui | 301·3(292·5-310·2) | 300·3(282·9-317·8) | -1·0(-9·2-7·3) | 0·9056 |
| Manchu | 292·2(282·6-301·7) | 309·3(303·6-315·0) | 17·2(12·0-22·4) | 0·0012 |
| Tibetan | 315·4(281·7-349·0) | 316·0(304·5-327·6) | 0·7(-15·1-16·4) | 0·9668 |
| Uighur | 258·7(253·8-263·6) | 253·2(242·1-264·2) | -5·5(-9·3--1·7) | 0·1491 |
| Zhuang | 333·0(325·9-340·2) | 334·6(325·1-344·1) | 1·6(-3·1-6·3) | 0·7367 |
| Others | 322·7(317·1-328·4) | 323·3(317·1-329·5) | 0·6(-2·8-4·0) | 0·8626 |

Values are mean (95%CI). All calculations including are weighted, accounting for the multistage cluster sampling design. CI=confidence interval. p values for difference refers to the comparison of 2015-16 and 2018-19.

# Supplementary Table 3: Prevalence of hyperuricemia diagnosed with different criteria in Chinese women aged 18 years and above, 2015-16 and 2018-19

|  | **2015-16** | |  | **2018-19** | |
| --- | --- | --- | --- | --- | --- |
|  | **Criteria 1** * | **Criteria 2** † |  | **Criteria 1** * | **Criteria 2** † |
| Total | 2·8(2·5-3·0) | 9·6(8·8-10·5) |  | 3·6(3·2-4·0) | 11·5(10·6-12·3) |
| Age groups (years) |  |  |  |  |  |
| 18-29 | 2·7(2·2-3·1) | 9·6(8·3-10·9) |  | 4·2(3·2-5·2) | 13·1(11·3-14·9) |
| 30-39 | 1·7(1·3-2·0) | 7·3(6·4-8·3) |  | 2·3(1·8-2·9) | 8·5(7·6-9·4) |
| 40-49 | 2·1(1·7-2·5) | 7·5(6·3-8·8) |  | 2·2(1·9-2·5) | 7·8(7·0-8·7) |
| 50-59 | 2·8(2·5-3·1) | 10·2(9·3-11·1) |  | 3·7(3·2-4·1) | 11·6(10·7-12·4) |
| 60-69 | 4·0(3·5-4·4) | 12·2(11·3-13·1) |  | 4·4(4·0-4·8) | 13·7(12·7-14·7) |
| ≥70 | 6·3(5·5-7·2) | 17·4(15·7-19·0) |  | 8·0(7·0-9·1) | 20·9(19·3-22·6) |

Values are % (95%CI). All calculations including are weighted, accounting for the multistage cluster sampling design. * Hyperuricemia was diagnosed with women>7 mg/dl (420 µmol/L). † Hyperuricemia was diagnosed with women>6 mg/dl (360 µmol/L).

# Supplementary Table 4: Means (µmol/L, 95%CI) of blood uric acid among men and women by sociographic characteristics, 2015-16 and 2018-19

|  | **Mean (µmol/L, 95%CI) of blood uric acid** | | **Changes of means, mean (µmol/L, 95%CI) 2018-19 vs 2015-16** | **p value for difference** |
| --- | --- | --- | --- | --- |
|  | **2015-16** | **2018-19** |  |  |
| **Men** |  |  |  |  |
| **Age group (years)** |  |  |  |  |
| 18-29 | 366·5(359·9-373·1) | 387·3(379·7-394·9) | 20·8(13·2-28·5) | <0·0001 |
| 30-39 | 357·9(354·0-361·9) | 376·9(371·8-381·9) | 18·9(14·3-23·5) | <0·0001 |
| 40-49 | 348·3(343·3-353·3) | 358·8(355·3-362·4) | 10·6(5·0-16·2) | <0·0001 |
| 50-59 | 338·5(335·0-342·1) | 347·4(343·9-350·8) | 8·8(4·1-13·6) | <0·0001 |
| 60-69 | 335·2(332·0-338·5) | 343·2(339·7-346·7) | 7·9(3·4-12·5) | <0·0001 |
| ≥70 | 341·9(336·4-347·4) | 349·7(346·1-353·4) | 7·9(1·3-14·4) | <0·0001 |
| **Residence** |  |  |  |  |
| Urban | 361·2(355·4-367·1) | 377·5(372·3-382·7) | 16·3(10·7-21·9) | <0·0001 |
| Rural | 340·9(337·9-344·0) | 352·6(348·7-356·5) | 11·7(7·6-15·8) | <0·0001 |
| **Education** |  |  |  |  |
| Primary school or less | 339·8(336·1-343·5) | 344·9(341·5-348·2) | 5·1(0·4-9·8) | 0·0334 |
| Secondary school | 347·4(343·4-351·5) | 357·9(354·1-361·7) | 10·5(5·9-15·0) | <0·0001 |
| High school | 362(353·6-370·4) | 379·1(372·2-386·1) | 17·1(9·7-24·5) | <0·0001 |
| College or higher | 370(365·5-374·5) | 396·9(390·3-403·5) | 26·9(19·4-34·4) | <0·0001 |
| **Ethnic** |  |  |  |  |
| Han | 351·3(346·9-355·8) | 366·2(361·9-370·5) | 14·9(10·8-19·0) |  |
| Hui | 344·3(332·9-355·6) | 346·7(325·1-368·2) | 2·4(-19·4-24·2) | 0·8283 |
| Manchu | 331·6(321·5-341·7) | 350·1(341·4-358·8) | 18·6(5·4-31·7) | 0·0058 |
| Tibetan | 373·2(330·5-415·9) | 364·0(349·3-378·7) | -9·2(-52·6-34·2) | 0·6759 |
| Uighur | 292·0(282·1-301·9) | 291·8(280·5-303·1) | -0·2(-7·1-6·7) | 0·9532 |
| Zhuang | 372·9(364·1-381·7) | 389·2(375·0-403·5) | 16·3(3·4-29·2) | 0·0135 |
| Others | 369·4(362·4-376·4) | 371·7(363·1-380·4) | 2·3(-9·0-13·6) | 0·6855 |
| **Women** |  |  |  |  |
| **Age group (years)** |  |  |  |  |
| 18-29 | 271·2(267·6-274·7) | 284·3(280·6-288·1) | 13·2(8·5-17·9) | <0·0001 |
| 30-39 | 259·2(256·5-262·0) | 267·7(264·9-270·5) | 8·4(5·2-11·7) | <0·0001 |
| 40-49 | 258·2(254·3-262·1) | 261·6(258·9-264·4) | 3·4(-1·3-8·1) | 0·1505 |
| 50-59 | 272·2(269·6-274·7) | 279·1(276·5-281·8) | 6·9(3·5-10·4) | <0·0001 |
| 60-69 | 278·0(275·1-280·8) | 283·6(281·0-286·2) | 5·6(2·2-9·0) | 0·0001 |
| ≥70 | 292·4(287·3-297·5) | 302·0(297·8-306·1) | 9·6(3·2-15·9) | 0·0032 |
| **Residence** |  |  |  |  |
| Urban | 272·5(268·8-276·3) | 282·6(279·6-285·7) | 10·1(6·1-14·1) | <0·0001 |
| Rural | 264·1(261·7-266·4) | 270·1(267·6-272·6) | 6·0(3·2-8·9) | <0·0001 |
| **Education** |  |  |  |  |
| Primary school or less | 268·8(265·9-271·7) | 274·8(272·0-277·7) | 6·1(2·7-9·4) | <0·0001 |
| Secondary school | 264·7(262·0-267·5) | 272·7(269·9-275·5) | 8·0(4·3-11·7) | <0·0001 |
| High school | 271·9(268·2-275·6) | 282·8(279·0-286·6) | 10·8(6·3-15·4) | <0·0001 |
| College or higher | 271·2(267·5-274·9) | 281·1(277·3-284·9) | 9·9(5·0-14·7) | <0·0001 |
| **Ethnic** |  |  |  |  |
| Han | 268·3(265·6-271·0) | 277·2(274·8-279·6) | 8·8(6·1-11·6) | <0·0001 |
| Hui | 266·0(255·6-276·4) | 261·2(246·6-275·8) | 4·8(-19·3-9·7) | 0·5152 |
| Manchu | 256·3(248·7-263·9) | 266·7(260·2-273·1) | 10·4(0·3-20·4) | 0·0433 |
| Tibetan | 271·1(248·8-293·4) | 270·3(261·9-278·7) | -0·8(-22·0-20·3) | 0·9397 |
| Uighur | 221·7(218·5-225·0) | 213·1(203·6-222·6) | -8·6(-20·1-2·9) | 0·1420 |
| Zhuang | 300·7(292·2-309·3) | 288·9(279·4-298·4) | -11·8(-23·1--0·6) | 0·0396 |
| Others | 277·2(270·9-283·5) | 279·3(274·6-283·9) | 2·1(-4·4-8·6) | 0·5326 |

Values are mean (95%CI). All calculations including are weighted, accounting for the multistage cluster sampling design. p value for difference refers to the comparison of 2015-16 and 2018-19. CI=confidence interval.

# Supplementary Table 5: Proportions of hyperuricemia by levels among Chinese adults by sociodemographic characteristics, 2015-16 and 2018-19

|  | **2015 -16** | | | |  | **2018-19** | | | | **ᵡ2** |  | ***p* value** |
| --- | --- | --- | --- | --- | --- | --- | --- | --- | --- | --- | --- | --- |
|  | **420-479µmol/L** | **480-539µmol/L** | **540-600µmol/L** | **>600µmol/L** |  | **420-479µmol/L** | **480-539µmol/L** | **540-600µmol/L** | **>600µmol/L** |  |  |  |
| **Overall** | 61·9(59·6-64·2) | 26·2(23·7-28·6) | 8·4(7·5-9·3) | 3·5(3·0-4·0) |  | 58·6(56·6-60·6) | 26·2(24·9-27.6) | 9·9(8·7-11·1) | 5·3(4·3-6·3) | 3·7571 |  | 0·0023 |
| **Sex** |  |  |  |  |  |  |  |  |  |  |  |  |
| Men | 60·9(58·3-63·4) | 26·9(24·2-29·7) | 8·7(7·7-9·7) | 3·5(3·0-4·1) |  | 56·5(54·3-58·7) | 27·2(25·7-28·7) | 10·6(9·2-11·9) | 5·7(4·6-6·9) | 4·0993 |  | 0·0006 |
| Women | 69·4(66·4-72·4) | 20·8(18·2-23·4) | 6·7(4·9-8·4) | 3·1(2·4-3·9) |  | 72·6(69·9-75·3) | 19·7(17·6-21·9) | 5·4(4·4-6·5) | 2·2(1·6-2·7) | 10·8169 |  | 0·1758 |
| **Age group (years)** |  |  |  |  |  |  |  |  |  |  |  |  |
| 18-29 | 58·8(53·0-64·6) | 29·4(22·8-36·1) | 8·0(5·8-10·2) | 3·8(2·6-4·9) |  | 58·0(53·6-62·3) | 24·5(20·9-28·0) | 11·1(7·4-14·8) | 6·4(4·5-8·4) | 4·6396 |  | 0·0657 |
| 30-39 | 63·0(57·9-68·0) | 25·6(21·8-29·3) | 8·3(6·2-10·4) | 3·1(2·0-4·2) |  | 56·6(53·3-59·9) | 28·8(25·9-31·7) | 9·9(8·0-11·7) | 4·7(2·9-6·5) | 1·0193 |  | 0·1093 |
| 40-49 | 64·3(60·7-67·8) | 24·3(22·0-26·7) | 8·6(6·1-11·0) | 2·9(2·0-3·7) |  | 59·1(56·0-62·3) | 27·4(24·8-29·9) | 8·7(7·2-10·3) | 4·8(3·7-5·8) | 1·6008 |  | 0·0711 |
| 50-59 | 63·4(60·9-65·9) | 24·9(22·9-27·0) | 8·2(6·5-9·8) | 3·5(2·6-4·4) |  | 61·1(58·8-63·4) | 25·6(23·7-27·5) | 9·2(7·9-10·5) | 4·1(3·3-4·9) | 0·8175 |  | 0·4809 |
| 60-69 | 64·0(61·7-66·2) | 23·0(21·2-24·8) | 8·7(7·5-9·9) | 4·4(3·3-5·4) |  | 60·7(59·0-62·5) | 24·6(23·2-25·9) | 9·1(8·1-10·2) | 5·6(4·7-6·5) | 2·1621 |  | 0·1355 |
| ≥70 | 59·7(57·0-62·4) | 26·0(23·7-28·3) | 10·0(8·5-11·5) | 4·3(3·3-5·2) |  | 60·0(57·0-62·9) | 25·5(23·4-27·6) | 9·5(8·0-11·0) | 5·0(3·9-6·2) | 3·1711 |  | 0·7793 |
| **Location** |  |  |  |  |  |  |  |  |  |  |  |  |
| Urban | 61·0(57·4-64·6) | 26·8(22·8-30·8) | 9·1(7·7-10·5) | 3·1(2·4-3·7) |  | 55·8(53·2-58·5) | 27·4(25·6-29·2) | 10·6(8·7-12·4) | 6·2(4·8-7·6) | 3·3514 |  | 0·0014 |
| Rural | 63·3(61·3-65·2) | 25·2(23·5-26·9) | 7·5(6·7-8·2) | 4·1(3·4-4·7) |  | 62·7(60·3-65·1) | 24·5(22·3-26·7) | 8·9(7·8-10·0) | 3·9(3·2-4·7) | 3·9257 |  | 0·3267 |
| **Education** |  |  |  |  |  |  |  |  |  |  |  |  |
| Primary school or less | 63·4(61·2-65·6) | 23·3(21·8-24·9) | 9·2(7·8-10·5) | 4·1(3·4-4·8) |  | 60·9(58·4-63·3) | 25·4(23·4-27·3) | 8·9(7·8-10·0) | 4·9(3·8-5·9) | 0·8018 |  | 0·3043 |
| Secondary school | 64·0(61·4-66·6) | 23·8(21·8-25·7) | 8·3(6·4-10·1) | 4·0(3·1-4·9) |  | 62·4(59·9-64·8) | 24·5(22·3-26·6) | 8·4(6·9-9·9) | 4·7(3·5-6·0) | 0·0867 |  | 0·7280 |
| High school | 53·4(44·5-62·2) | 35·6(24·9-46·3) | 8·8(6·5-11·2) | 2·2(1·3-3·1) |  | 54·9(49·8-60·0) | 27·8(24·3-31·2) | 12·3(7·7-16·9) | 5·0(2·7-7·4) | 13·0887 |  | 0·0605 |
| College or higher | 66·2(62·7-69·7) | 23·4(20·5-26·3) | 7·2(5·1-9·3) | 3·2(2·1-4·3) |  | 55·1(50·8-59·4) | 27·9(23·7-32·1) | 10·5(7·6-13·3) | 6·6(4·4-8·8) | 2·8394 |  | 0·0009 |
| **Ethnic** |  |  |  |  |  |  |  |  |  |  |  |  |
| Han | 62·1(59·6-64·6) | 26·2(23·5-28·9) | 8·3(7·3-9·2) | 3·4(2·9-3·9) |  | 58·4(56·2-60·5) | 26·3(24·9-27·7) | 10·0(8·7-11·2) | 5·4(4·4-6·4) | 3·1164 |  | 0·0016 |
| Hui | 55·3(41·1-69·6) | 37·6(23·5-51·8) | 2·7(0·4-4·9) | 4·4(0·1-8·6) |  | 53·3(41·1-65·6) | 31·1(23·1-39·1) | 12·3(2·3-22·2) | 3·3(0·8-5·9) | 6·5475 |  | 0·0412 |
| Manchu | 61·8(54·4-69·2) | 19·2(15·2-23·3) | 12·8(6·4-19·2) | 6·1(2·1-10·2) |  | 74·5(63·6-85·4) | 20·5(11·0-30·1) | 3·6(0·9-6·3) | 1·4(0·0-3·0) | 15·9620 |  | 0·0005 |
| Tibetan | 62·3(55·2-69·4) | 25·6(20·8-30·4) | 7·3(5·3-9·2) | 4·8(3·4-6·3) |  | 73·3(70·1-76·5) | 18·4(16·4-20·5) | 6·4(3·6-9·2) | 1·9(0·5-3·3) | 3·2843 |  | <0·0001 |
| Uighur | 75·5(69·5-81·5) | 13·2(1·9-24·4) | 6·7(0·0-13·6) | 4·7(0·0-13·1) |  | 86·6(74·9-98·3) | 6·2(0·0-15·5) | 3·9(0·0-9·7) | 3·3(0·0-9·4) | 2·1791 |  | 0·7378 |
| Zhuang | 66·3(57·5-75·2) | 27·3(16·0-38·5) | 4·5(0·6-8·4) | 1·9(0·7-3·1) |  | 55·0(40·6-69·4) | 34·4(21·0-47·9) | 5·5(3·1-7·8) | 5·1(2·0-8·1) | 3·6458 |  | 0·3601 |
| Others | 58·3(54·5-62·1) | 26·0(22·8-29·2) | 11·6(8·6-14·6) | 4·1(3·1-5·1) |  | 56·6(52·7-60·5) | 25·4(20·9-29·9) | 13·0(11·1-15·0) | 5·0(3·0-6·9) | 1·6414 |  | 0·7466 |
| **Income per capita (CNY)** |  |  |  |  |  |  |  |  |  |  |  |  |
| Q1 (<6000) | 64·0(60·4-67·6) | 24·8(21·3-28·3) | 7·7(6·8-8·7) | 3·5(2·6-4·3) |  | 63·3(58·9-67·7) | 22·9(19·0-26·9) | 9·6(7·6-11·6) | 4·2(2·6-5·7) | 3·0073 |  | 0·4757 |
| Q2 (6000-) | 63·6(60·0-67·1) | 25·6(22·5-28·8) | 6·8(5·4-8·1) | 4·1(2·8-5·3) |  | 58·4(52·5-64·4) | 27·3(23·0-31·7) | 8·5(6·2-10·9) | 5·7(2·7-8·7) | 2·5868 |  | 0·3261 |
| Q3 (12000-) | 62·6(60·0-65·2) | 25·2(22·2-28·1) | 8·9(6·6-11·2) | 3·3(2·5-4·1) |  | 58·9(54·9-62·9) | 25·6(22·3-28·9) | 9·2(7·4-11·0) | 6·3(4·6-8·0) | 2·3980 |  | 0·0264 |
| Q4 (24000-) | 58·7(52·3-65·1) | 29·4(21·3-37·6) | 8·8(6·6-11·0) | 3·0(2·0-4·0) |  | 59·8(55·9-63·7) | 25·7(23·1-28·3) | 9·4(7·7-11·2) | 5·1(3·3-7·0) | 4·1479 |  | 0·2883 |
| Refused/Don’t know | 63·1(58·7-67·4) | 23·7(20·2-27·1) | 9·3(5·3-13·3) | 4·0(2·9-5·1) |  | 54·1(49·0-59·2) | 28·8(25·8-31·8) | 12·4(7·6-17·1) | 4·7(3·6-5·9) | 0·5473 |  | 0·1043 |

Values are % (95%CI). All calculations including are weighted, accounting for the multistage cluster sampling design. p value refers to the comparison of two survey years. CI=confidence interval.
